# Fibroblast Growth Factor—14 Acts as Tumor Suppressor in Lung Adenocarcinomas

**DOI:** 10.3390/cells9081755

**Published:** 2020-07-22

**Authors:** Kati Turkowski, Frederik Herzberg, Stefan Günther, David Brunn, Andreas Weigert, Michael Meister, Thomas Muley, Mark Kriegsmann, Marc A. Schneider, Hauke Winter, Michael Thomas, Friedrich Grimminger, Werner Seeger, Soni Savai Pullamsetti, Rajkumar Savai

**Affiliations:** 1 Max Planck Institute for Heart and Lung Research, 61231 Bad Nauheim, Germany; kati.turkowski@mpi-bn.mpg.de (K.T.); frederik.herzberg@mpi-bn.mpg.de (F.H.); stefan.guenther@mpi-bn.mpg.de (S.G.); david.brunn@mpi-bn.mpg.de (D.B.); werner.seeger@mpi-bn.mpg.de (W.S.); soni.pullamsetti@mpi-bn.mpg.de (S.S.P.); 2 Institute of Biochemistry I, Faculty of Medicine, Goethe University Frankfurt, 60590 Frankfurt, Germany; weigert@biochem.uni-frankfurt.de; 3 Frankfurt Cancer Institute (FCI), Goethe University, 60596 Frankfurt am Main, Germany; 4 Translational Research Unit, Thoraxklinik at Heidelberg University, 69126 Heidelberg, Germany; michael.meister@med.uni-heidelberg.de (M.M.); thomas.muley@med.uni-heidelberg.de (T.M.); marc.schneider@med.uni-heidelberg.de (M.A.S.); 5 Translational Lung Research Center (TLRC) Heidelberg, 69126 Heidelberg, Germany; Mark.kriegsmann@med.uni-heidelberg.de (M.K.); hauke.winter@med.uni-heidelberg.de (H.W.); michael.thomas@med.uni-heidelberg.de (M.T.); 6 Institute of Pathology, University Hospital of Heidelberg, 69126 Heidelberg, Germany; 7 Department of Surgery, Thoraxklinik, University Hospital Heidelberg, 69126 Heidelberg, Germany; 8 Department of Oncology, Thoraxklinik, University Hospital Heidelberg, 69126 Heidelberg, Germany; 9 Department of Internal Medicine, Justus Liebig University, 35392 Giessen, Germany; friedrich.grimminger@innere.med.uni-giessen.de; 10 Institute for Lung Health (ILH), Justus Liebig University, 35392 Giessen, Germany

**Keywords:** fibroblast growth factor 14, lung adenocarcinoma, xenograft model, lung cancer mesenchymal epithelial transition

## Abstract

Investigation of the molecular dynamics in lung cancer is crucial for the development of new treatment strategies. Fibroblast growth factor (FGF) 14 belongs to the FGF family, which might play a crucial role in cancer progression. We analyzed lung adenocarcinoma (LUAC) patients samples and found that FGF14 was downregulated, correlating with reduced survival and oncogenic mutation status. FGF14 overexpression in lung cancer cell lines resulted in decreased proliferation, colony formation, and migration, as well as increased expression of epithelial markers and a decreased expression of mesenchymal markers, indicating a mesenchymal to epithelial transition in vitro. We verified these findings using small interfering RNA against FGF14 and further confirmed the suppressive effect of FGF14 in a *NOD*.*Cg*-*Prkdc^scid^* Il2rg^tm1Wjl^/*SzJ* immunodeficient xenograft tumor model. Moreover, FGF14 overexpressing tumor cell RNA sequencing data suggests that genes affected by FGF14 were related to the extracellular matrix, playing a role in proliferation and migration. Notably, newly identified FGF14 target genes, adenosine deaminase RNA specific B1 (*ADARB1*), collagen and calcium-binding epidermal growth factor domain-containing protein 1 (*CCBE1*), α1 chain of collagen XI (*COL11A1*), and mucin 16 (*MUC16*) expression was negatively correlated with overall survival when *FGF14* was downregulated in LUAC. These findings led us to suggest that FGF14 regulates proliferation and migration in LUAC.

## 1. Introduction

Lung cancer is one of the leading global causes of cancer associated deaths worldwide [1] and is responsible for 15.7–23.5% of all cancer-associated deaths in women and men, respectively, in Germany [2]. In 2016, 21,500 women and 35,960 men were newly diagnosed with lung cancer in Germany, with a 5-year-survival rate of 21% and 15% for women and men. As a heterogeneous disease, lung cancer is traditionally classified as either small cell lung cancer (SCLCs) or non-small cell lung cancer (NSCLC) [3]. NSCLCs are further classified as various subtypes using both histological and molecular techniques [3,4]. Lung adenocarcinomas (LUAC) (42%) and squamous cell carcinoma (LUSC) (20%) are the most commonly diagnosed NSCLC subtypes [2]. The types of genetic alterations and the clinical outcomes differ according to the subtype [4], particularly LUAC as an oncogene driven cancer type [5]. While the accumulation of mutations and epigenetic abnormalities leads to the expression of multiple genes with diverse functions, a better understanding of the genomic landscape in cancer provides more prospects for NSCLC treatment based on the tumor molecular characteristics [6]. 

The fibroblast growth factor (FGF) family comprises 22 genes [7,8], and there is evidence that some members play a role in oncogenic mechanisms such as proliferation, survival, angiogenesis, migration, and invasion [9]. Phylogenetically, FGFs can be classified into three groups that interact with different co-factors. Both canonical and endocrine FGFs can bind to one of five corresponding tyrosine-kinases receptors (FGFRs) [7,8]. FGF11, FGF12, FGF13, and FGF14 belong to a noncanonical subset of intracellular FGFs [7,8,10]. Those factors are also known as fibroblast growth factor homologous factors (FHFs), and although they have a high structural and sequence similarity, FHFs are unable to activate FGFRs [10,11]. In contrast to other FGFs, FHFs lack an N-terminal secretion sequence, and it has been suggested that FHFs act as intracellular signaling molecules [12,13]. Fibroblast growth factor (FGF) signaling plays an important role in tumors and was found to accompany >1000 somatic mutations found in the coding exons of 518 protein kinase genes from 210 different human cancers [14]. Additionally, a study of 623 sequenced genes in 188 cases of primary LUAC revealed that the fibroblast growth factor receptor (FGFR) family was highly dysregulated in 19% of the cases [15]. Ongoing clinical trials targeting FGF in NSCLC are mainly pursuing FGFR signaling [16,17]. Therefore, further investigation of the molecular dynamics of FGF signaling in NSCLCs is crucial. 

In healthy tissues, FGF14 is primarily expressed in the thymus, testicles, and brain [18]. In the brain, FGF14 is highly expressed during embryogenesis and interacts with various ion-channels [18,19,20,21]. Loss of FGF14 function is associated with spinocerebellar ataxia type 27, a very rare subtype of type I autosomal dominant cerebellar ataxia and psychiatric disorders [7,22]. Moreover, FGF14 is expressed in two isoforms FGF14-a and FGF14-b [18]. FGF14-b is primarily located in the cytoplasm, and FGF14-a can bind to DNA and is found in the nucleus [18,23]. FGF14 expression is altered in various tumors [24,25,26] and it has been suggested to regulate apoptosis and proliferation in nasopharyngeal carcinoma and colorectal cancer [26,27]. Nonetheless, the role of FGF14 in lung cancer, especially in NSCLC remains unelucidated. This study investigated the role of FGF14 in NSCLC using in vitro and in vivo experiments to examine its biological function. We further analyzed the downstream signaling targets of FGF14 to determine the molecular mechanism of its biological function in NSCLC tumorigenicity.

## 2. Materials and Methods

### 2.1. Cell Culture

A549 cells (ATCC CCL-185, American Type Culture Collection, Manassas, VA, USA) were cultured in Dulbecco’s Modified Eagle Medium Nutrient Mixture F12 (Gibco, Life Technologies, Carlsbad, CA, USA) containing 10% fetal bovine serum (Gibco) and 1% of penicillin-streptomycin (10,000 U/mL, Gibco). H838 cells (ATCC CRL-5844, American Type Culture Collection) and H460 cells (ATCC-HTB-177, American Type Culture Collection) were cultured in Roswell Park Memorial Institute medium 1640, Gibco) containing 10% of FBS and 1% of penicillin-streptomycin (10,000 U/mL, Gibco). All cell lines were cultured at 37 °C under 5% CO_2_ and passaged every 2–3 days when reaching confluence.

### 2.2. Transfection

A549 and H838 cells were seeded in a 96-well-plate (5 × 10^4^ cells/well) and incubated for 24–48 h for the overexpression of FGF14. For a 4-well transfection, 200 µL of a transfection solution containing 1 µg FGF14-plasmid (RC219002, OriGene Technologies, Rockville, MD, USA), 6 µL Fugene HD transfection reagent (Promega, Madison, WI, USA), and Opti-MEM reduced serum medium (Gibco) was prepared. After 24 h, the medium was changed to each cell line’s culture medium, to which 10 µL/mL Geniticin (G418 Sulfate) (Gibco) was added for selecting successfully transfected cells. Subsequently, the cell number increased, and the cells were assayed. For small interfering RNA (siRNA) transfection, 1 × 10^5^ A549, H838 and H460 cells were seeded in 6-well plates and incubated for 48 h. Then, the cells were transfected with ON-TARGETplus human FGF14 siRNA or non-targeting control siRNA (Horizon, Cambridge, UK) using DharmaFECT transfection reagent (GE Healthcare, Chicago, IL, USA), according to the manufacturer’s instructions. Finally, the cells were harvested and assayed 48–72 h post transfection.

### 2.3. Real-Time Polymerase Chain Reaction (qPCR)

RNA was extracted from cells by Chomczynski and Sacchis’s single-step-method using Trizol reagent (Ambion, Life Technologies, Carlsbad, CA, USA). An miRNeasy Mini Kit (Qiagen, Hilden, Germany) was used as per the manufacturer’s instructions for siRNA-transfected cells. cDNA was synthesized using a High Capacity cDNA Reverse Transcription Kit (Thermo Fisher Scientific, Waltham, MA, USA). For RT-PCR, cDNA was amplified in triplicates using the *StepOnePlus* Real-Time PCR System (Applied Biosystems, Foster City, CA, USA) using PowerUP SYBR Green Master Mix (Thermo Fisher, Waltham, MA, USA). The following primers were used: *18S*: Forward (5′-GGCCCTGTAATTGGAATGAGTC-3′), Reverse (5′-CCAAGATCCAACTACGAGCTT-3′); *FGF14* Forward (5′-GGAAGGGCAAGCTATGAAAGG-3′), Reverse (5′-TGGTTCTCGGTACATGGCAAC-3′); *COL11A1* Forward (5′-ACCCTCGCATTGACCTTCC-3′), Reverse (5′-TTTGTGCAAAATCCCGTTGTTT-3′); *ADARB1* Forward (5′-GTGAAGGAAAACCGCAATCTGG-3′), Reverse (5′-CAGGAGTGTGTACTGCAAACC-3′); *CCBE1* Forward (5′- CGACTAAATACCCGTGTCTGAAG-3′), Reverse (5′-TCGGCACAAACGTCGTAATCT-3′); *MUC16* Forward (5′-CCAGTCCTACATCTTCGGTTGT-3′), Reverse (5′-AGGGTAGTTCCTAGAGGGAGTT-3′). The *18S* rRNA gene was used as the housekeeping gene. The delta cycle (dCT) was calculated as follows: average (CT (*18S*))–average (CT (GOI)).

### 2.4. Western Blot

Total protein was extracted from cells using RIPA lysis-buffer (Santa Cruz Biotechnology, Dallas, TX, USA). A bicinchoninic acid (BCA) assay (Bio-Rad Laboratories, Hercules, CA, USA) was performed to determine the protein concentration. After dilution of the protein concentration to 1 µg/mL, the proteins were separated by sodium dodecyl sulfate-polyacrylamide-gel-electrophoresis (Bio-Rad Laboratories) and blotted using a wet electroblotting system (Bio-Rad Laboratories) on a polyvinylidene difluoride-membrane (Bio-Rad Laboratories). Then, the membranes were blocked with 5% skim milk in tris-buffered saline with Tween (TBST) and incubated with primary antibodies overnight at 4 °C. The following primary antibodies were used: FGF14 (1:1000, ab229610), Caspase8 (1:1000, ab52183), mouse β-actin (1:5000, ab6276), and Cyt18 (1:1000, ab52948) from Abcam (Cambridge, UK) and Caspase7 (1:1000, #9492), Claudin-1 (1:1000, #4933), CDH2 (1:1000, #14215) and β-catenin (1:1000, #9582) from Cell Signaling Technology (Danvers, MA, USA). Subsequently, the membranes were washed in Tris-buffered saline with Tween20 (TBST) and incubated for 2 h with a corresponding horseradish peroxidase (HRP)-conjugated secondary antibody, either anti-rabbit HRP IgG Conjugate (1:3000; Promega, Madison, WI, USA) or anti-mouse HRP IgG Conjugate (1:3000, Promega). The resultant chemiluminescence signals were detected after treatment with Western Bright Sirius chemiluminescent substrate (Advansta, Menlo Park, CA, USA) using ImageQuant (GE Healthcare, Chicago, IL, USA).

### 2.5. Colony Formation Assay

For the colony formation assay, approximately 300 cells/well of each cell clone (empty vector (EV)/expression vector FGF14-OE) were seeded in triplicate in a 6-well plate and incubated in 10% FBS medium for 7 days. Thereafter, the cells were washed with phosphate-buffered saline (PBS). After fixation in methanol for 3 min, cells were stained using 10% crystal violet (Sigma-Aldrich, St Louis, Missouri, USA) for 5 min. Photographs were taken, and the colonies were counted using ImageJ’s cell counter (Fiji).

### 2.6. Bromodeoxyuridine (BrdU) Assay

We used a colorimetric immunoassay to quantify cell proliferation (Cell Proliferation ELISA, BrdU (colorimetric); Roche, Basel, Switzerland), according to manufacturer’s instructions. Briefly, each cell clone (EV/FGF14-OE) was seeded into 16 wells (2 rows) of a 96-well plate at a concentration of 5 × 10^4^ cells/well and starved overnight in medium containing 0% FBS. The next day, the medium in 8 wells was changed to 10% FBS. The colorimetric signals were detected using a microplate reader (Tecan, Männedorf, Switzerland) to measure the optical density (OD) at 370 and 492 nm. The % proliferation was calculated as follows: FGF14-OE (OD_370 nm_ − OD_492 nm_)/average EV ([OD_370 nm_ − OD_492 nm_] × 100).

### 2.7. Scratch Assay

For the scratch assay, each cell clone (EV/FGF14-OE) was seeded at a concentration of 1 × 10^5^ cells/well in 4 wells of a 24-well plate and grown to a confluency of >90%. Then, the cells were scratched using a micropipette tip and washed with PBS. Following the addition of 1% FBS medium to each well, the cells were incubated for 18 h. The scratches were photographed (5× magnification) at 0 h and 18 h of incubation. The scratch areas were calculated using the area function of ImageJ (Fiji). The % of the scratch closed by cell growth was calculated as follows: 100—(area [18 h] / area [0 h] *100). Four replicates per condition were conducted for each experiment (*n* = 3).

### 2.8. Migration Assay (Boyden Cöhamber)

To perform the cell migration assay, 5 × 10^5^ cells/well of each cell clone (EV/FGF14-OE) were resuspended in FBS-free medium and seeded into 24-well cell culture inserts (Falcon, Corning, New York, NY, USA). The lower chamber was filled with medium containing 10% FBS. Due to the migratory capacity of the used cells lines, incubation time was adapted (A549: 5–6 h, H838: 16 h, and H460: 24 h). After incubation, the inner side of the membrane of the cell culture inserts was cleared from cells using a cotton swap. Then, the membranes were fixed with methanol for 3 min and stained with crystal violet for 10 min (Sigma-Aldrich). The membranes were cut out using a scalpel, placed on a slide, and covered with Pertex mounting medium (Medite Service AG, Dietikon, Switzerland). The slides were scanned using a NanoZoomer slide scanner (Hamamatsu Photonics, Hamamatsu, Japan). We quantified the number of migratory cells per membrane using ImageJ software with the plug-in ITCN (National Institutes of Health, Bethesda, MD, USA). The % migration was calculated as follows: detected peaks [FGF14-OE]/average (detected peaks [EV]) *100. Four technical replicates were conducted for each experiment.

### 2.9. Immunocytochemistry (ICC)

Immunocytochemical analyses were performed by seeding 1 × 10^4^ cells/well in an 8-well chamber slide (SARSTEDT, Nürnbrecht, Germany), followed by incubation for 24–48 h prior to staining. Briefly, the cells were fixed for 15 min by using 4% paraformaldehyde, washed with PBS (Gibco), treated with Triton-X for 15 min, and blocked with 1% bovine serum albumin (BSA)/PBS solution for 30 min. The primary antibodies FGF14 (1:300, ab229610), Cyt18 (1:200, ab52948) from Abcam (Cambridge, UK) and Claudin-1 (1:100, #4933), CDH2 (1:100, #14215) and β-catenin (1:100, #9582) from Cell Signaling Technologies, Danvers, MA, USA), were diluted in 1% BSA/PBS solution and incubated with the cells for 2 h. After washing the cells with 1% BSA/PBS solution for 15 min, Alexa Flour 488 goat anti-rabbit (A11008 Invitrogen, Thermo Fisher Scientific, Waltham, MA, USA) was used as the secondary antibody, and the cells were incubated for 90 min while protected from light. Thereafter, the chambers were removed, and the slide was washed three times with PBS. The Nuclei were counterstained using Immunoselect Antifading Mounting Medium DAPI (DIANOVA, Hamburg, Germany). All slides were stored at 4 °C, and photographs were acquired at constant exposure and gain by fluorescent microscopy (Leica, Wetzlar, Germany).

### 2.10. Transcriptome Analyses

For RNA-seq, as previously described [28], we isolated RNA from A549-FGF14 overexpressing cells and empty vector control cells. The statistical significance of differentially expressed genes (DEGs) were analyzed based on a combination of absolute expression (basemean), divergence (log2 fold change, log2fc), and significance (adjusted *p*-value, padj). The *p*-value denotes the probability that results are significant (i.e., significantly different based on absolute counts and divergence). Furthermore, the *p*-value identifies genes/peaks with reasonable expression rates and fold changes that show reproducible counts between replicates of conditions. The terms padj, false discovery rate (FDR), and Q-value are not consistently defined, but generally denote a *p*-value after the application of a multiple testing correction (e.g., Benjamini–Hochberg) to control for the number of false positives. These are limited to 5% if padj is set to 0.05. Thus, if there are 20 candidates with padj <0.05, only one of them will be a false-positive result. If the *p*-value was used without correction, the number of false positives is statistically unknown. Intuitively, multiple testing correction was performed because it was easier to find the only 10 correct candidates among 100 genes than among 1,000,000 genes, since every test increases the chance of obtaining a false-positive result. The log2FC is calculated as follows: log2(mean [counts condition 1])—log2(mean [counts condition 2]). Fragments per kilobase of transcript per million mapped reads was used to normalize the sequencing depth and gene length to permit the comparison of results between genes and samples as follows: 10^9^ × gene count/ (mappable reads in total of the sample × gene length). The Z-score was defined as the number of standard deviations that a value is above or below the mean of all values.

### 2.11. Human Lung Tissues Samples

RNA samples from human LUAC tissue were obtained from the Lungbiobank Heidelberg, member of the Biomaterialbank Heidelberg and the Biobank platform of the German Center for Lung Research. Lung tissue specimens embedded in paraffin were obtained from the Institute for Pathology (Giessen, Germany). The study protocol for tissue donation was approved by the Ethics Committee (“Ethik-Kommission des Fachbereichs Medizin der Justus-Liebig-Universität Giessen”) of the University Hospital Giessen (Giessen, Germany) in accordance with the national law and “Good Clinical Practice/International Conference on Harmonisation” guidelines. Written informed consent was provided by each patient or the patients next of kin (AZ 58/15).

### 2.12. Mouse Model

*NOD*.*Cg*-*Prkdc^scid^* Il2rg^tm1Wjl^/*SzJ* (NSG) mice were purchased from Charles River Laboratories (Sulzfeld, Germany). A549-EV and A549-FGF14-OE cells (1 × 10^6^) were randomly injected subcutaneously into the right flank of 6–8-week-old NSG mice as previously described [29,30]. Based on the protocol, the animals were sacrificed after 40 days of tumor growth for further analyses (*n* = 7). All experiments using animal models were performed according to the German Law for Animal Protection and the National Institute of Health Guidelines for Care and Use of Laboratory Animals, which were approved by local authorities (Regierungspräsidium Darmstadt, Hessen, Germany, Approval number B2/1202).

### 2.13. Immunohistochemistry (IHC)/Immunofluorescence Staining

In preparation for IHC staining, 3-µm tissue sections were rehydrated, and antigen-retrieval was achieved with citrate buffer as described elsewhere [31]. Then, the sections were blocked and incubated with rabbit anti-FGF14 (1:100) primary antibody overnight. A ZytoChem Plus AP Polymer Kit (Zytomed Systems, Berlin, Germany) was used for detection, following the manufacturer’s instructions. Sections were embedded with Pertex and scanned using a NanoZoomer slide scanner (Hamamatsu Photonics). For immunofluorescence staining, cryosections were incubated with 0.3% Triton-X in PBS for 10 min, followed by blocking with 1% BSA in PBS for 1 h at room temperature. The primary antibodies FGF14 (1:300, ab229610), CYK18 (1:200, ab52948) from Abcam (Cambridge, UK) and Claudin-1 (1:100, #4933), CDH2 (1:100, #14215) and β-catenin (1:100, #9582) from Cell Signaling Technologies, Danvers, MA, USA), which were diluted in a 1% BSA/PBS solution and incubated with the cells for 2 h. After washing the sections 3 times with 1% BSA/PBS solution for 10 min, Alexa Flour 488 goat anti-rabbit was used as the secondary antibody. The sections were incubated for 90 min while protected from light. Thereafter, they were washed 3 times with PBS. The Nuclei were counterstained using Immunoselect Antifading Mounting Medium DAPI. All sections were stored at 4 °C, and photographs were acquired at constant exposure and gain by fluorescent microscopy (Leica) under 40× magnification.

### 2.14. Data and Statistical Analyses

Statistical significance was determined using GraphPad Prism 8 software (GraphPad Software, San Diego, CA, USA) and the two-tailed unpaired or paired (where applicable) Student’s *t*-test as well as one-way or two-way analysis of variance (ANOVA), as indicated in the relevant figure legends. Comparisons of 2 experimental groups with 1 control group were calculated using one-way ANOVA with Dunnett’s multiple comparisons test. Data is shown as mean ± standard error of the mean (SEM) of at least 3 independent experiments unless otherwise stated. Datasets used by CANCERTOOL: http://cbio.mskcc.org/public/lung_array_data/ and GSE31210; (The Cancer Genome Atlas, TCGA).

## 3. Results

### 3.1. FGF14 Downregulation Is Associated with Poor Overall Survival (OS) of NSCLC Patients

**Figure 1:**
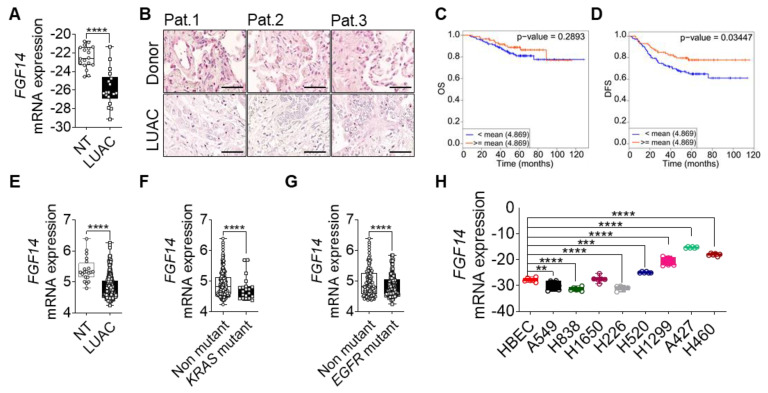
Clinical outcome associated with *FGF14* expression in lung adenocarcinoma (LUAC). (**A**) mRNA expression analysis of FGF14 in LUAC patients samples (*n* = 18) compared to non-tumor tissue (NT). (**B**) Immunohistochemical staining of FGF14 in LUAC and healthy donor tissue samples (*n* = 3). Scale bar 50 µm. Kaplan–Meier estimate of (**C**) overall survival (OS) and (**D**) disease free survival (DFS) among Okayama patients dataset with LUAC that was classified according to the levels of *FGF14* mRNA as either high (above the mean value of *FGF14* mRNA levels; red) and low (below the mean value of *FGF14* mRNA levels; blue). (**E**) mRNA expression level of *FGF14* in LUAC samples from the same study compared with non-tumor tissue. (**F**) Analysis of *FGF14* expression in *KRAS* mutant vs *KRAS* non-mutant patients samples and (**G**) *EGFR* mutant vs *EGFR* non-mutant samples. (**H**) mRNA expression of *FGF14* in different lung cancer cell lines e.g., LUAC (A549, H1299, H838, and H1650), LUSC (H226. and H520), lung carcinoma (A427) and large cell lung cancer (H460) compared with primary human bronchial epithelial cells (HBEC). Data obtained from CANCERTOOL (**C**–**G**). Data shown as mean+/- standard error of the mean using Student’s *t*-test. *P*-values ≤ 0.05 were considered statistically significant for all analyses. ** *p* ≤ 0.01, *** *p* ≤ 0.001 and **** *p* ≤ 0.0001.

We investigated the role of FGF14 in lung cancer progression by first analyzing the mRNA expression of *FGF14* in lung cancer samples from patients with LUAC. We found that FGF14 expression at mRNA and protein level was significantly reduced compared with samples from non-tumor or healthy donor lung tissue (Figure 1A,B). Furthermore, we confirmed our findings by analyzing existing data from lung cancer patient cohorts using CANCERTOOL software [32]. The data revealed that *FGF14* expression was negatively correlated with the patients overall survival (OS) (Figure 1C) and disease-free survival (DFS) (Figure 1D) in the Okayama patients cohort. Survival curves from two other data sets (Chitale, TCGA) also indicated the same trend for OS (Figure S1A,B), metastasis free survival (MFS) (Figure S1C), and DFS (Figure S1D). Moreover, mRNA expression level of *FGF14* was significantly downregulated in this patients cohort (Figure 1E). In addition, data from patients with mutations in the *KRAS* (Figure 1F) and *EGFR* (Figure 1G) oncogenes showed a significant decrease in *FGF14* expression correlating with reduced overall survival. It appeared that *FGF14* expression was reduced in the later stages of lung cancer and differed between the data sets (Figure S1E,F). We also analyzed the DNA methylation of *FGF14* in a cohort of LUAC patients using UCSC Xena Platform [33]. Both, the 27 k and 450 k Illumina Methylation Assay data sets (Figure S1G,H) showed a significant increase of *FGF14* DNA-methylation in LUAC samples compared with non-tumor tissue. However, the OS of these patients was unaffected (Figure S1I,J). We also examined the expression of *FGF14* in human lung cancer cell lines. In comparison with human bronchial epithelial cells (HBEC), the *FGF14* expression varied between the cell lines.

While *FGF14* expression was less in some LUAC cell lines (A549, H838, H1299, and H1650), it differed in squamous cell carcinoma (LUSC) cell lines (H226 and H520). In contrast, *FGF14* was highly expressed in a large cell carcinoma cell line (H460) and lung carcinoma (A427) (Figure 1H). Moreover, we validated *FGF14* expression in several cancer cell lines, breast (MCF-7), prostate (DU145, PC3) ovary (SKOV3, OVCAR3), liver (HEP2G), pancreatic (MiaPaCa-2, Capan-1), and colon (Colo320, SW480), revealing a substantial variability (Figure S1K). Kaplan–Meier curves showed [34] negative correlation of patients OS harboring breast, ovary, or liver cancer (Figure S1L–N).

### 3.2. Overexpression of FGF14 in Tumor Cells Suppresses Proliferation, Colony Formation, and Migration, and Changes Mesenchymal to Epithelial Transition in NSCLC Cells

**Figure 2:**
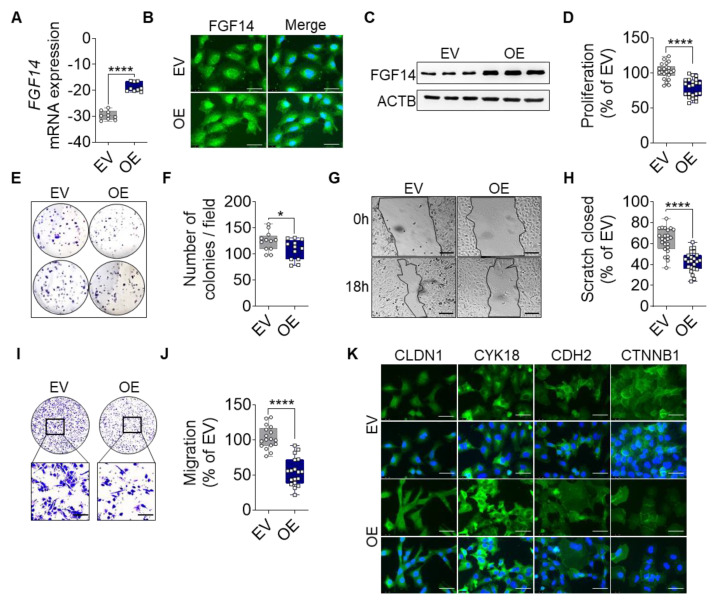
FGF14 overexpression reveals a tumor-suppressive phenotype in vitro. Validation of FGF14 overexpression after transfection of A549 cells with empty vector (EV) and FGF14 expression vector (OE) was quantified by (**A**) qRT-PCR, (**B**) immunocytochemistry (ICC) staining and (**C**) Western blot. Cells in B panel were labeled using FGF14 antibody and revealed by an AlexaFlour 488 secondary antibody (green). DNA was stained with 4′,6-diamidino-2-phenylindole (blue), scale bars, 50 µm. (**D**) Cellular proliferation was quantified by BrdU incorporation of FGF14 OE cells compared to EV control cells. (**E**,**F**) Colony formation of FGF14 OE cells compared with EV control cells. The migratory ability of FGF14 OE cells was assessed via (**G**,**H**) scratch assay and (**I**,**J**) Boyden chamber assay. Representative pictures were taken at 0 and 18 h after scratching; scale bars, 100 µm. (**K**) ICC staining of epithelial (CLDN1, CYK18) and mesenchymal marker (CDH2, CTNNB1) labeled using an AlexaFlour 488 secondary antibody (green). DNA was stained with 4′,6-diamidino-2-phenylindole (blue), scale bars, 50 µm. Data are shown as mean ± standard error of the mean using Student’s *t*-test. *P*-values ≤ 0.05 were considered statistically significant for all analyses. (*n* = 3) * *p* ≤ 0.05 and **** *p* ≤ 0.0001.

Based on the above findings, we overexpressed FGF14 in human LUAC cell lines to investigate the role of this gene in lung cancer progression. A549 cells were transfected with a vector carrying FGF14 (OE) or an empty vector (EV) as control. The overexpression was verified at the mRNA level (Figure 2A) as well as at the protein level (Figure 2B,C).

The measurement of proliferation via BrdU incorporation revealed significantly less proliferation of FGF14 OE cells compared with the EV control (Figure 2D). To determine the behavior of the FGF14 overexpressing cells, we analyzed the ability of the cells to form colonies (Figure 2E,F). We found a significantly reduced number of colonies by FGF14 OE cells in comparison with EV control cells. Migration was assessed using scratch assay (Figure 2G,H) and Boyden chamber assay (Figure 2I,J). In both cases a significantly reduced migratory behavior was observed compared with the EV control cells. Moreover, based on our previous studies regarding EMT [30,35], we examined the expression of epithelial and mesenchymal markers (Figure 2K). Here, we found an increase in epithelial marker expression (CLDN1, Claudin1; CYK18, Cytokeratin18) and a decrease of mesenchymal markers (CDH2, N-cadherin; and CTNNB1, β-Catenin) by FGF14 overexpressing cells compared with the EV control indicating that these cells were in the process of mesenchymal and epithelial transition. In addition, we used the same experimental setup and also detected these phenotypes in other lung cancer cell lines H838 (Figure S2A–H) and H460 (Figure S3A–F), which have a high basal expression of FGF14. Collectively, these findings demonstrate that FGF14 overexpression markedly decreased the invasive potential of lung cancer cells in vitro.

### 3.3. Genetic Ablation of FGF14 in Tumor Cells Revert Phenotypic Changes and Supports a Suppressive Role in NSCLC Cells

**Figure 3:**
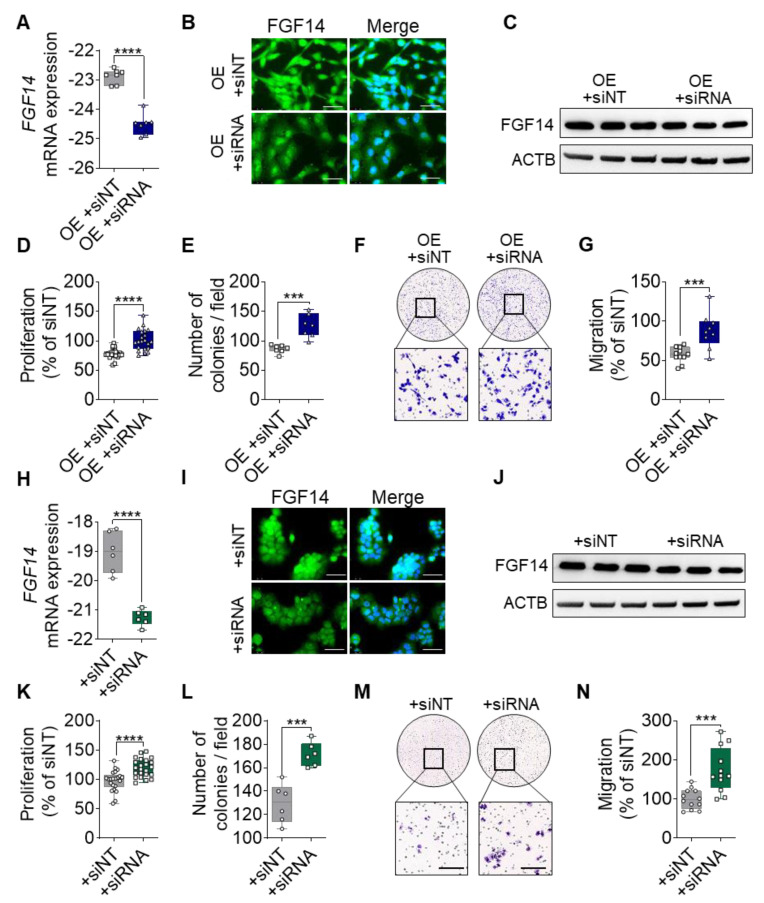
Silencing of FGF14 abrogate the suppressive phenotype in FGF14 overexpressing A549 cells. (**A**) mRNA expression of *FGF14* after siRNA transfection with FGF14 siRNA and non-targeting siRNA control (siNT). (**B**) ICC images of FGF14 after treatment with FGF14 siRNA compared with non-targeting control. Cells were labeled by using antibody against FGF14 and detected using AlexaFlour488 secondary antibody (green). Nuclear DNA was counterstained with DAPI (blue), scale bars, 50 µm. (**C**) Western blotting of FGF14 OE cells after treatment with FGF14 siRNA. (**D**) Cellular proliferation by BrdU incorporation, (**E**) colony formation and (**F**,**G**) Boyden chamber migration was performed after treatment of FGF14 OE cells with siNT compared to FGF14 siRNA. (**H**) mRNA expression level of *FGF14* in H460 cells after siRNA treatment. (**I**) Representative pictures of FGF14 ICC staining of siRNA and non-targeting siRNA treated H460 cells. (**J**) Western blotting of H460 cells after siRNA transfection. In vitro assays including (**K**) proliferation, (**L**) colony formation and (**M**,**N**) migration to determine functional changes upon FGF14 silencing. Data are shown as mean ± standard error of the mean using Student’s *t*-test (*n* = 3). *P*-values ≤0.05 were considered statistically significant for all analyses. *** *p* ≤ 0.001 and **** *p* ≤ 0.0001.

To prove that the suppressive phenotype is due to the overexpression of FGF14 and not a secondary effect, we silenced the expression of FGF14 in the A549-FGF14OE cells and H460 cells. After transfection of the FGF14 overexpressing cells with siRNA against *FGF14* (OE+siRNA), we detected the significantly decreased expression of *FGF14* at the mRNA level compared with the non-targeting control transfected cells (OE+siNT) (Figure 3A). The reduced level of protein expression was also observed via ICC staining (Figure 3B) and Western blotting (Figure 3C). Furthermore, there was a significantly increased proliferation and colony formation of FGF14-overexpressing A549 cells post siRNA transfection compared with the NT control siRNA treated cells (Figure 3D,E). Additionally, we evaluated the same behavior in the Boyden chamber migration assay, where a significant increase in migration was observed following FGF14 siRNA treatment (Figure 3F,G). To restrain these findings, we performed additional experiments with H460 cells, a large cell lung cancer cell line that has a high baseline expression of FGF14. Following FGF14 siRNA treatment, we detected a significant decrease in *FGF14* expression on mRNA level (Figure 3H) as well as protein level (Figure 3I,J). The functional assays revealed a significant increase of proliferation, colony formation, and migration compared with the NT siRNA transfected cells (Figure 3K–N). Collectively, these experiments confirm that FGF14 contribute to the decreased proliferation and migration.

### 3.4. Overexpression of FGF14 Reduces Tumor Progression in a Subcutaneous Xenograft Tumor Model

To investigate the tumor suppressive properties of FGF14 overexpressing cells in vivo, we transplanted A549-FGF14 OE and A549-EV cells subcutaneously into the right flank of *NOD*.*Cg*-*Prkdc^scid^* Il2rg^tm1Wjl^/*SzJ* (NSG) immunodeficient mice.

**Figure 4:**
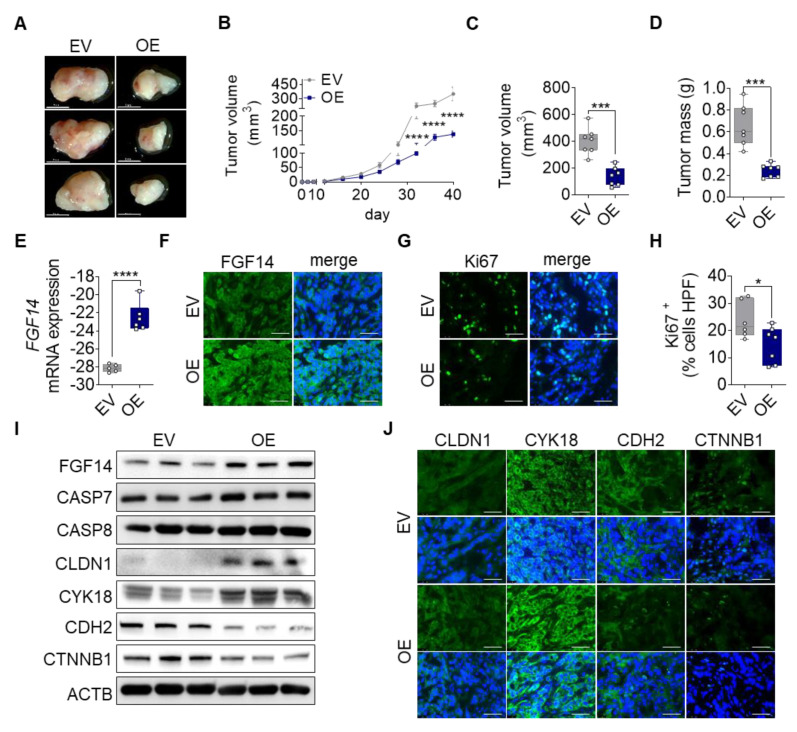
Impact of FGF14 overexpression on cancer progression in vivo. A549-EV and A549-FGF14 OE cells were injected in the right flank of immunodeficient mice. Tumors were harvested after 40 days. (**A**) Representative photographs of dissected FGF14 overexpressing tumors. (**B**) Measurement of tumor volume during tumor progression. Quantification of (**C**) tumor volume and (**D**) tumor mass after tumor dissection. Revalidation of FGF14 expression in mice tumor samples via (**E**) qPCR and (**F**) immunohistochemistry staining. (**G**) Ki67 staining of proliferating cells within the tumor were (**H**) counted per high power field using ImageJ (Fiji) Software. Representative pictures of FGF14 and Ki67 staining visualized using Alexa Flour 488 coupled secondary antibody (green). (**I**) Validation of FGF14 overexpression, apoptosis using antibodies against CASP7 and CASP8 and epithelial marker expressions using antibodies against CLDN1 and CYK18 and mesenchymal marker expressions evaluated by antibodies against CDH2 and CTNNB1. (**J**) Additional immunohistochemistry staining of epithelial and mesenchymal marker. Representative pictures of epithelial markers (CDH1 and CYK18) and mesenchymal markers (CDH2 and CTNNB1) visualized using Alexa Flour 488 coupled secondary antibody (green). Nuclear DNA was counterstained with DAPI (blue), scale bar 50 µm. Data shown as mean ± standard error of the mean using one-way analysis of variance (*n* = 7). *P*-values ≤ 0.05 were considered statistically significant for all analyses. * *p* ≤ 0.05, *** *p* ≤ 0.001, and **** *p* ≤ 0.0001.

Mice injected with A549-FGF14 OE cells showed already macroscopic reduction of tumor volume compared with the EV control (Figure 4A). The tumor progression was measured using calipers every fourth day until 40 days. From the time of inoculation, the tumor volume over time showed decreased tumor progression in FGF14 OE cells injected animals (Figure 4B). These findings were confirmed by quantifying the total tumor volume (401.8 ± 38.35 mm^3^ vs 137.4 ± 26.21 mm^3^) and tumor weight (0.66 ± 0.07 g vs 0.24 ± 0.02 g) (Figure 4C,D). Notably, we could detect FGF14 overexpression in the tumor tissue after 40 days of tumor growth at the mRNA (Figure 4E) and protein level (Figure 4F,I). Furthermore, we observed a significantly decreased number of proliferating cells in the tissue samples via quantification of the Ki67 positive cells (Figure 4G,H). In addition, apoptosis was increased in these tumors visualized by Western blotting (Figure 4I) detecting increased protein band size for CASP7 and CASP8 respectively. Moreover, Western blotting (Figure 4I) and immunofluorescence staining (Figure 4J) of tumor tissue revealed changes in epithelial and mesenchymal marker expression. While epithelial markers (CDH1 and CYK18) were upregulated in A549-FGF14 overexpressing tumors, mesenchymal markers (CDH2, CTNNB1) showed a decreased expression indicating MET, and this confirmed our previous findings in vitro.

### 3.5. FGF14 Overexpression Alters Gene Expression Profile

**Figure 5:**
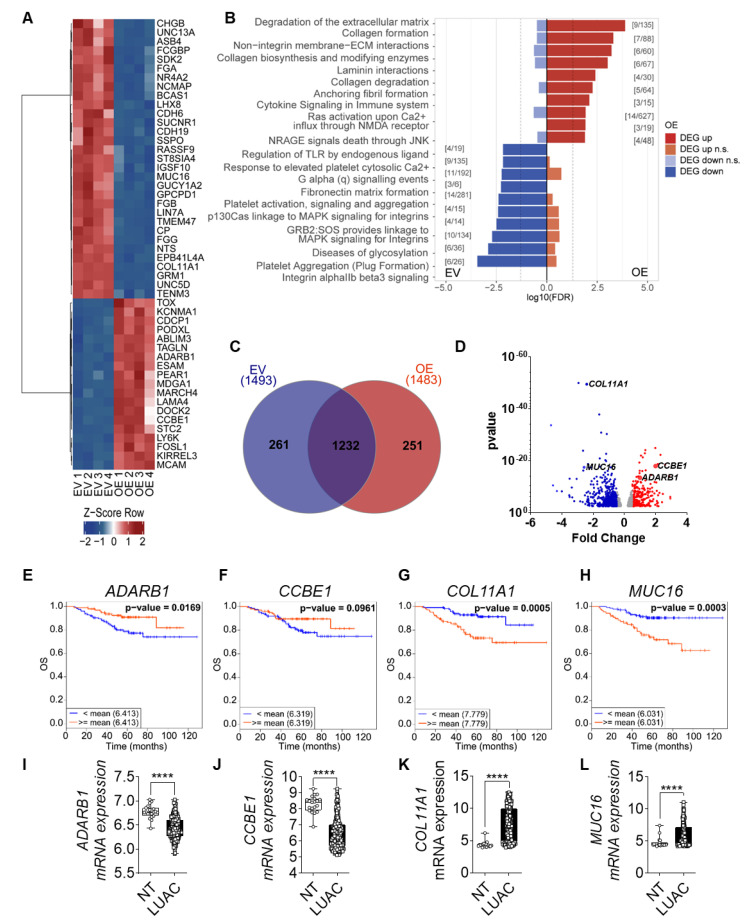
FGF14 overexpression impacts gene expression profile of tumor cells. (**A**) Heatmap of top 50 most significant differentially expressed genes (DEGs) for each contrast (sorted by smallest adjusted *p*-value). (**B**) The DEGs, split into up and downregulated genes, a gene set enrichment analysis was performed using KEGG Orthology-Based Annotation System (KOBAS). The graph represents the top 10 gene sets or pathways enriched for up- or downregulated genes of one database (dashed line: *p*-value = 0.05). The graph only shows pathways that were not significant for both directions (up and down). Significant gene set enrichment was defined by the false discovery rate (FDR). (**C**) Venn diagram of DEGs using InteractiVenn [36]. (**D**) Volcano plot: bottom track = *p*-value based definition of a DEG (less stringent, only for comparison). (**E**–**H**) Kaplan–Meier estimate of OS among the Okayama patients dataset with LUAC classified according to the mRNA expression levels of *CCBE1, ADARB1, COL11A1*, and *MUC16* as either high (above the mean value of mRNA levels, red) and low (below the mean value of mRNA levels, blue). (**I**–**L**) mRNA expression level of in LUAC samples from the same study compared with non-tumor tissue. Data was obtained from CANCERTOOL. Data are presented as mean ± standard error of the mean using Student’s *t*-test. *p*-values ≤ 0.05 were considered statistically significant for all analyses **** *p* ≤ 0.0001.

We further investigated FGF14 targets by performing mRNA sequencing analysis of A549-FGF14 overexpressing cells versus A549-EV transfected cells. The bioinformatics analysis revealed that a variety of genes were up or downregulated in A549-FGF14 OE cells. The top 50 DEGs were plotted on a heatmap (Figure 5A). In addition, gene enrichment analysis of the sequencing data revealed regulation of genes related to the extracellular matrix (Figure 5B). We constructed a Venn diagram depicting the number of DEGs in A549-EV cells versus A549-FGF14 OE cells (Figure 5C). Interestingly, the genes involved in the extracellular matrix were upregulated (*CCBE1,* and *ADARB1*) and downregulated (*COLL11A1,* and *MUC16*), as highlighted in the volcano plot (Figure 5D). Furthermore, *CCBE1* and *ADRB1* which were upregulated in FGF14 overexpressing cells were associated with a decrease in OS (Figure 5E,F) and DFS (Figure S4A,B) when downregulated in LUAC patients. The opposite was observed for the decreased expression of *COL11A1* and *MUC16* in A549-FGF14 overexpressing cells, where the upregulation of these genes was associated with a decrease in OS (Figure 5G,H) and DFS (Figure S4C,D). The expression analysis of existing datasets from CANCERTOOL showed a significant downregulation of *CCBE1* and *ADARB1* (Figure 5I,J), and a significant upregulation of *COL11A1* and *MUC16* (Figure 5K,L) in samples from LUAC patients. Moreover, the *KRAS* (Figure S4E–H) and *EGFR* (Figure S4I–L) mutation status for these LUAC patients was not strongly affected. The expression of *ADARB1* was significantly downregulated in *KRAS* mutant samples (Figure S4E). The expression of *CCBE1* and *MUC16* showed a significant reduction in *EGFR* mutant samples (Figure S4J,L) compared with *EGFR* non-mutant samples.

### 3.6. FGF14 Target Gene Expression in Human LUAC Tissues and FGF14 Overexpressing and Silencing Samples Confirming Transcriptomic Findings

**Figure 6:**
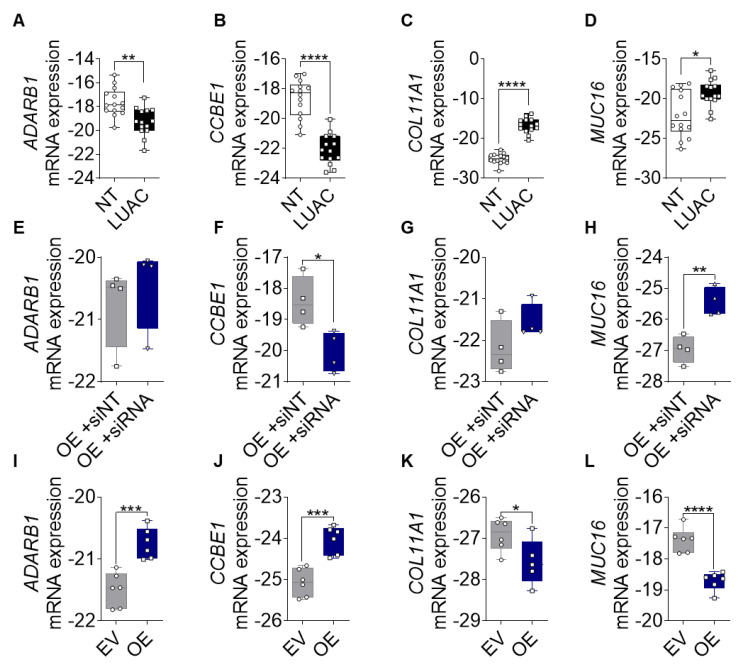
Validation of FGF14 target genes. (**A**–**D**) mRNA expression of *ADARB1, CCBE1, COL11A1*, and *MUC16* in human LUAC tissue compared with samples from non-tumor tissue (*n* = 14). (**E**–**H**) mRNA expression silencing of FGF14 in A549-FGF14 OE cells and non-targeting siRNA control samples (*n* = 4). (**I**–**L**) mRNA expression of FGF14 target genes in FGF14 OE and FGF14 EV tumor tissues from immunodeficient xenograft model (*n* = 6). Data are presented as mean ± standard error of the mean using Student’s *t*-test. *P-*values ≤ 0.05 were considered statistically significant for all analyses. * *p* ≤ 0.05, ** *p* ≤ 0.01, *** *p* ≤ 0.001, and **** *p* ≤ 0.0001.

Based on the transcriptomic data results we aimed to confirm the expression of newly identified FGF14 target genes (*ADRAB1, CCBE1, COL11A1*, and *MUC16*) in different sample types. First, we analyzed the expression of these genes in samples from LUAC patients. The expression differed between samples of lung tumor tissue and the non-tumor tissue sample from the same patient. The mRNA expression of *CCBE1* and *ADARB1* was significantly upregulated (Figure 6A,B), while the expression of *COL11A1* and *MUC16* (Figure 6C,D) was significantly downregulated in LUAC samples compared with non-tumor samples. To evaluate whether expression of these genes could be restored after FGF14 silencing, we transfected the A549-FGF14 OE cells with non-targeting siRNA as well as FGF14 siRNA respectively. We found a significant downregulation of *CCBE1* and upregulation of *MUC16* compared with the NT siRNA control transfected cells (Figure 6F,H), while the effect of silencing for the targets *ADARB1* and *COL11A1* was not significant compared with the NT siRNA control (Figure 6E,G). We also examined the expression of the FGF14 target genes in samples obtained from FGF14 overexpressing xenograft tumors. In contrast with the human samples, *ADARB1* and *CCBE1* were upregulated (Figure 6I,J) and *COL11A1* and *MUC16* (Figure 6K,L) were downregulated in FGF14 overexpressing xenograft tumors, which emphasized the tumor suppressing role of FGF14 in NSCLC.

## 4. Discussion

The present study provides strong evidence that FGF14 plays a role in regulating proliferation- and migration-related genes, thereby suppressing NSCLC progression. Therefore, the targeting of FGF14 may provide new therapeutic approaches in terms of tumor growth inhibition, supported by the following findings. First, FGF14 correlated with decreased survival of NSCLC patients. Second, FGF14 overexpression leads to a suppressive phenotype in vitro that can be abolished by FGF14 silencing in NSCLC cells. Third, FGF14 overexpression reduces tumor progression in subcutaneous tumors in vivo. Last, downstream target genes of FGF14 are associated with NSCLC proliferation and migration.

As a part of this network, FGF/FGFR signaling is involved in a variety of cellular processes, including proliferation and migration [9]. FGF14, as a member of the non-secreted type FGF family, lacks many amino acid residues that are critical for the binding of FHF to the receptors [37]. There are several studies on FGF14 in regard to brain disorders; it is known to interact with voltage gated sodium channels [22]. However, barely anything in the context of other diseases, such as lung cancer, is known. In fact, whenever FGF14 was downregulated, such as in LUAC, colorectal, and nasopharyngeal cancer, it correlates with decreased OS [26,27,38]. A recent study on FGF14 in colorectal cancer reported tumor suppressive properties [26], which falls in line with our findings. The authors suggest that transcriptional silencing of *FGF14* was regulated by DNA-methylation. In silico analysis of a LUAC patients cohort (TCGA) using the UCSC Xena Platform [33] also showed an increased DNA methylation in tumor tissue compared with normal tissue. Nevertheless, this increase in methylation does not affect the OS for these patients. Despite the methylation, FGF14 expression could also be influenced by genetic alterations, like the oncogenes *KRAS* and *EGFR*, which both play a pivotal role in NSCLC. Analysis of the Okayama patients cohort revealed that *FGF14* is even more downregulated in patients with *KRAS* and *EGFR* mutantions compared with patients with non-mutant *KRAS* and *EGFR,* and this might be a hint that FGF14 expression is dependent on oncogene mutations. Additionally, adenocarcinoma cell line A549, was a known *KRAS* mutated cell line and matches with data from patients with mutated *KRAS,* also showing *FGF14* downregulation.

Therefore, to exclude the possibility that the impact of FGF14 was not mutation-related, we overexpressed FGF14 in the LUAC cell line A549, known to be *KRAS* mutated, and the non-mutant H838 cell line. Tumor cell functional results from in vitro assays, e.g., reduced proliferation and colony formation, which fall in line with prior studies on colorectal cancer and nasopharyngeal carcinoma [26,27]. We further proved the direct effect of tumor cell functional changes due to FGF14 overexpression and were able to abrogate the suppressive properties of FGF14 by siRNA treatment of the NSCLC cells. Based on our findings and the results of the other studies on FGF14 in the context of colorectal cancer and nasopharyngeal carcinoma, there is a strong evidence that FGF14 plays a role in tumor cell proliferation, migration, and invasion [26,27]. The loss of polarity and disruption of cell-cell adhesion indicates that EMT is one of the critical programs of malignant cancer cells for invasion and metastatic spread [39]. The induction of mesenchymal to epithelial transition (MET) by reprogramming the malignant phenotype of the cancer cell seems to be induced by overexpression of FGF14. However, at the metastatic site, it was postulated that MET also plays a role in the process of metastatic tumor formation, especially in the later stages of metastasis [40,41]. For instance, MET was reported by Oltean and colleagues among lung micro-metastases in a prostate cancer model [42]. How MET takes place and how it facilitates the formation of metastases, is largely unknown and has to be investigated intensively.

To further investigate the tumor suppressive properties of FGF14 in vivo, we used a *NOD*.*Cg*-*Prkdc^scid^* Il2rg^tm1Wjl^/*SzJ* xenograft model and observed approximately 65 % reduction in tumor volume and mass in FGF14 overexpressing tumors compared with the control tumors. A similar effect of FGF14 overexpression on tumor size was recently shown in Balb/c nude mice in a colorectal cancer model [26]. The investigators showed an induction of apoptosis due to FGF14 overexpression [26], congruent with our findings in the subcutaneous xenograft tumors. Despite the changes of epithelial and mesenchymal markers that we also found in the tumors, the metastatic potential in vivo could not be confirmed by this xenograft model and must be further investigated using an established mouse model for metastatic spread.

As malignant lung cancer cells are characterized by uncontrolled proliferation and migration programmed by an altered cancer transcriptome [43], the regulatory network that controls a wide range of pathological processes during cancer progression and metastasis is not fully understood. The identification of FGF14 target genes via mRNA sequencing of A549-FGF14 overexpressing cells versus A549-EV control cells gave us an additional hint concerning the involvement of FGF14 in proliferation and migration by gene set enrichment analysis. *ADARB1*, one of the upregulated genes due to FGF14 overexpression, was downregulated in LUAC patients and shows a negative correlation with OS and DFS. Additionally, patients with mutated *KRAS* showed a significant decrease of *ADARB1* expression compared with non-mutant patients. These data are consistent with those of a recent study on *ADARB1* in LUAC. The investigators also showed an increased migratory capacity after treatment of the cells with siRNA against *ADARB1* [44]. Other studies on glioblastoma multiforme and pediatric astrocytoma indicated that *ADARB1* overexpression leads to decreased proliferation of U87 cells [45] and decreased proliferation and migration of A172 and U118 cells [46]. *CCBE1* was another interesting gene that was upregulated upon FGF14 overexpression, and it was associated with decreased OS and DFS in LUAC patients when downregulated. Moreover, when *EGFR* was mutated, this gene was significantly downregulated. As in NSCLC, *CCBE1* is also diminished in ovarian cancer [47] and breast cancer [48]. *CCBE1* overexpression decreases colony formation and migration in T-47D human breast cancer cells [47]. In addition to an increased expression, some genes were also downregulated by the overexpression of FGF14, such as *COL11A1* which encodes the α1 chain of collagen XI and *MUC16*, a well-established biomarker used to monitor the progression and recurrence of ovarian cancer [49]. As components of the extracellular matrix these genes are involved in cell proliferation, migration, and invasion in several cancers, such as ovarian, gastric, and pancreatic as well as NSCLC [50,51,52,53,54,55]. The upregulation of these genes correlates with poor OS in patients LUAC patients. We were able to confirm the increased mRNA expression of *COL11A1* and *MUC16* in samples from LUAC patients compared with non-tumor lung tissue samples from the same patient. We detected the downregulation of *COL11A1* and *MUC16* in A549-FGF14 overexpressing cells, which was abrogated via treatment with the corresponding siRNA, suggesting that *COL11A1* and *MUC16* might be direct targets of FGF14. However, the mechanism underlying the manner in which FGF14 regulates the genes in NSCLC remains undetermined and requires elucidation. In summary, FGF14 is downregulated in LUAC, which was correlated with poor overall survival. We demonstrated that the FGF14 overexpression leads to a suppressive phenotype of NSCLC cells accompanying by diminished proliferation, colony formation, and migration in vitro and a reduced tumorgenicity in vivo. Our results show that FGF14 plays a role in the regulation of these cellular processes. Therefore, newly established inhibitors targeting the tumor suppressive properties of FGF14 would be a promising therapeutic strategy for LUAC patients and need further investigation to identify the underlying molecular mechanisms, thereby enabling the development of new therapeutic options.

## Supplementary Materials

The following are available online at https://www.mdpi.com/2073-4409/9/8/1755/s1. Figure S1: In silico comparative analyses of FGF14 mRNA expression in lung, breast, ovary and liver cancers; Figure S2: FGF14 overexpression in H838 cells; Figure S3: FGF14 expression in H460 cells; Figure S4: Kaplan–Meier estimate of overall survival in lung adenocarcinoma patients.
