# Supplementary material for: Fibroblast Growth Factor—14 Acts as Tumor Suppressor in Lung Adenocarcinomas
Source: Cells. 2020 Jul 22;9(8):1755. doi: 10.3390/cells9081755 (PMC7466013; doi:10.3390/cells9081755)
Supplement: Supplementary file 1 [file cells-09-01755-s001.pdf]

cells

Supplementary material

Fibroblast growth factor–14 acts as tumor suppressor in lung adenocarcinomas

Kati Turkowski[1], Frederik Herzberg[1], Stefan Günther[1], David Brunn[1], Andreas Weigert[2,3], Michael Meister[4,8], Thomas Muley[4,8], Mark Kriegsmann[7,8], Marc A. Schneider[4,8], Hauke Winter[5,8], Michael Thomas[6,8], Friedrich Grimminger[9], Werner Seeger[1,9], Soni Savai Pullamsetti[1,9], and Rajkumar Savai[1,3,9,10]*

[1]Max Planck Institute for Heart and Lung Research, Member of the German Center for Lung Research (DZL), Member of the Cardio-Pulmonary Institute (CPI), Bad Nauheim, 61231, Germany; kati.turkowski@mpi-bn.mpg.de (K.T.); frederik.herzberg@mpi-bn.mpg.de (F.H.); stefan.guenther@mpi-bn.mpg.de (S.G.); david.brunn@mpi-bn.mpg.de (D.B.); soni.pullamsetti@mpi-bn.mpg.de (S.P.); werner.seeger@mpi-bn.mpg.de (W.S.); rajkumar.savai@mpi-bn.mpg.de (R.S.)

[2]Institute of Biochemistry I, Faculty of Medicine, Goethe University Frankfurt, Frankfurt, 60590, Germany; weigert@biochem.uni-frankfurt.de (A.W.)

[3]Frankfurt Cancer Institute (FCI), Goethe University, 60596, Frankfurt am Main, Germany

[4]Translational Research Unit, Thoraxklinik at Heidelberg University, member of the DZL, 69126 Heidelberg, Germany; michael.meister@med.uni-heidelberg.de (M.M.); thomas.muley@med.uni-heidelberg.de (T.M.); marc.schneider@med.uni-heidelberg.de (M.S.)

[5]Dept. of Surgery, Thoraxklinik, University Hospital Heidelberg, 69126 Heidelberg, Germany; hauke.winter@med.uni-heidelberg.de (H.W.)

[6]Dept. of Oncology, Thoraxklinik, University Hospital Heidelberg, 69126 Heidelberg, Germany; michael.thomas@med.uni-heidelberg.de (M.T.)

[7]Inst. of Pathology, University Hospital of Heidelberg, 69126 Heidelberg, Germany; Mark.kriegsmann@med.uni-heidelberg.de (M.K.)

[8]Translational Lung Research Center (TLRC) Heidelberg, member of the German Center for Lung Research (DZL), 69126 Heidelberg, Germany

[9]Department of Internal Medicine, Member of the DZL, Member of CPI, Justus Liebig University, 35392, Giessen, Germany; friedrich.grimminger@innere.med.uni-giessen.de (F.G.)

[10]Institute for Lung Health (ILH), Justus Liebig University, 35392, Giessen, Germany

***Correspondence:** rajkumar.savai@mpi-bn.mpg.de; Tel.: +49 6032 - 705420

Content:

Supplementary Figure S1: *In silico* comparative analyses of FGF14 mRNA expression in lung, breast, ovary and liver cancers.

Supplementary Figure S2: FGF14 overexpression in H838 cells.

Supplementary Figure S3: FGF14 expression in H460 cells.

Supplementary Figure S4: Kaplan–Meier estimate of overall survival in lung adenocarcinoma patients.

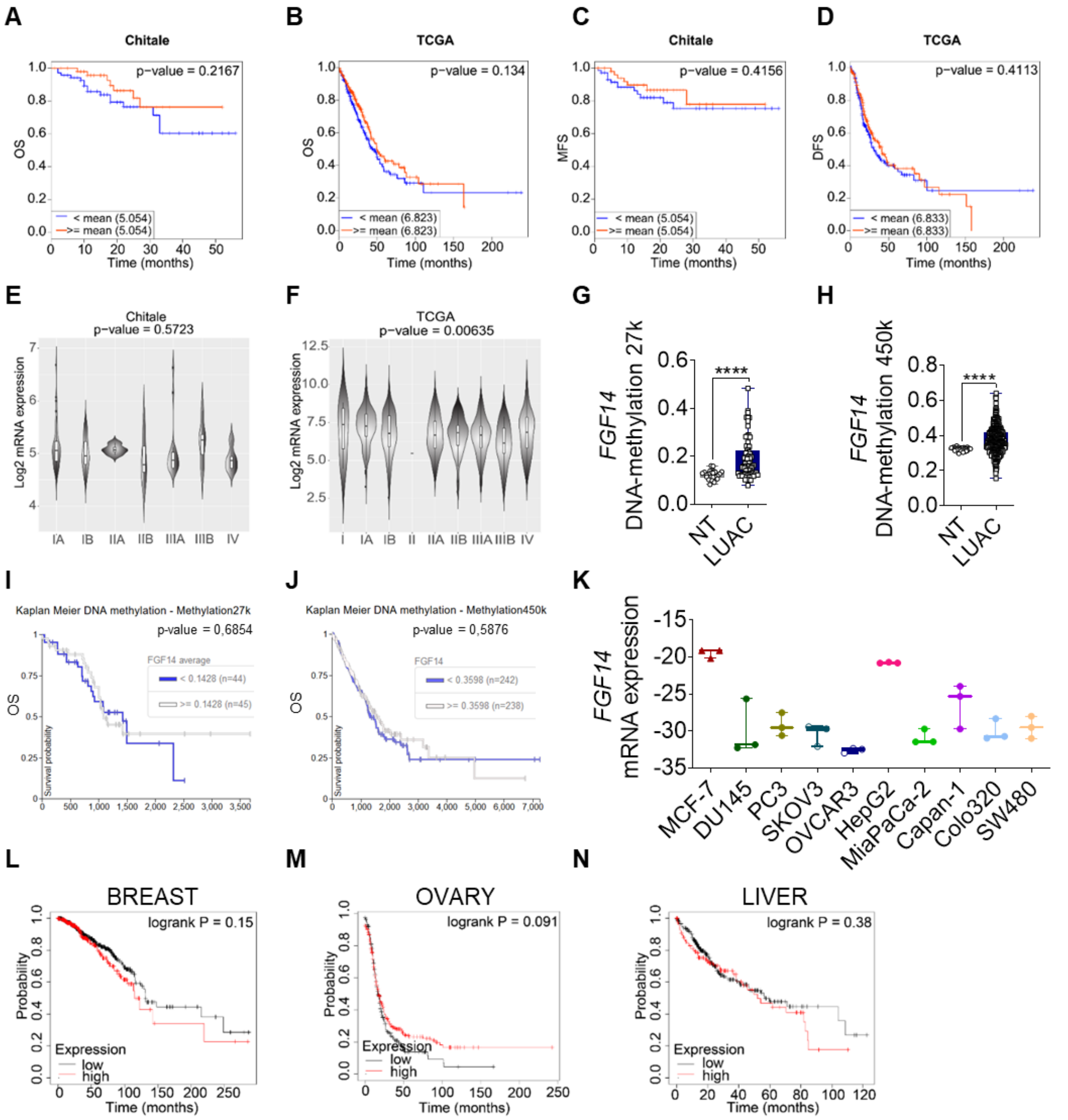

Figure S1. *In silico* comparative analyses of FGF14 mRNA expression in lung, breast, ovary and liver cancers. (**A-F**) Graphics include Kaplan–Meier curves representing overall survival (OS) (**A, B**), metastasis-free survival (MFS) (**C**), and disease-free survival (DFS) (**D**) of patient groups selected according to the expression of *FGF14* in the datasets designated as indicated above each graphic. (**E–F**) Violin plots depict the expression of FGF14 among cancer specimens of the indicated group of tumors in the different datasets. The pathological stage is indicated as IA, IB, II, IIA, IIB, IIIA, IIIB, or IV. An analysis of variance test was performed to compare the mean among groups [32]. FGF14-DNA methylation (**G**) 27k and (**H**) 450k in LUAC compared with non-tumor tissue. (**I-J**) OS of LUAC patients according to FGF14-DNA methylation. (**K**) *FGF14* mRNA expression in different cancer cell lines, such as breast (MCF-7), prostate (DU145 and PC3) ovary (SKOV3 and OVCAR3), liver (HEP2G), pancreatic (MiaPaCa-2 and Capan-1), and colon (Colo320 and SW480 (**L–N**). Adjusted *p*-values were calculated using the Benjamini–Hochberg (BH) method. Methylation data (**G–J**) was obtained from The Cancer Genome Atlas LUAC cohort using Xena browser [33].

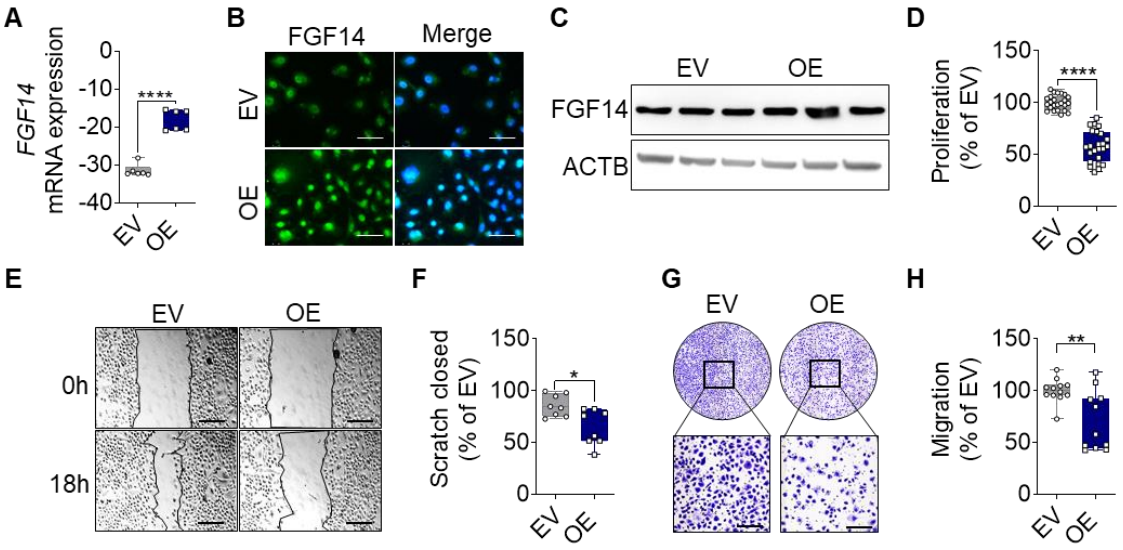

Figure S2. FGF14 overexpression in H838 cells. Validation of FGF14 overexpression by H838 cells via (**A**) real-time polymerase chain reaction, (**B**) immunocytochemistry staining, and (**C**) western blot. (**D**) Cellular proliferation was quantified by bromodeoxyuridine incorporation in H838-FGF14-expression vector (OE) cells compared with H838-empty vector (EV) control cells. The migratory ability of FGF14-OE cells was assessed via scratch assay (**E, F**) and Boyden chamber assay (**G, H**) compared with the EV control cells. Representative photographs were taken at 0 and 18 h after scratching (5× magnification). The cells in B were labeled using FGF14 antibody and revealed using an AlexaFlour 488 secondary antibody (green). DNA was stained with 4′,6-diamidino-2-phenylindole (blue), scale bars, 100 μm. Data are shown as mean± standard error of the mean using Student's t-test. *P*-values ≤ 0.05 were considered statistically significant for all analyses. *$p \le 0.05$, **$p \le 0.01$, and ****$p \le 0.0001$.

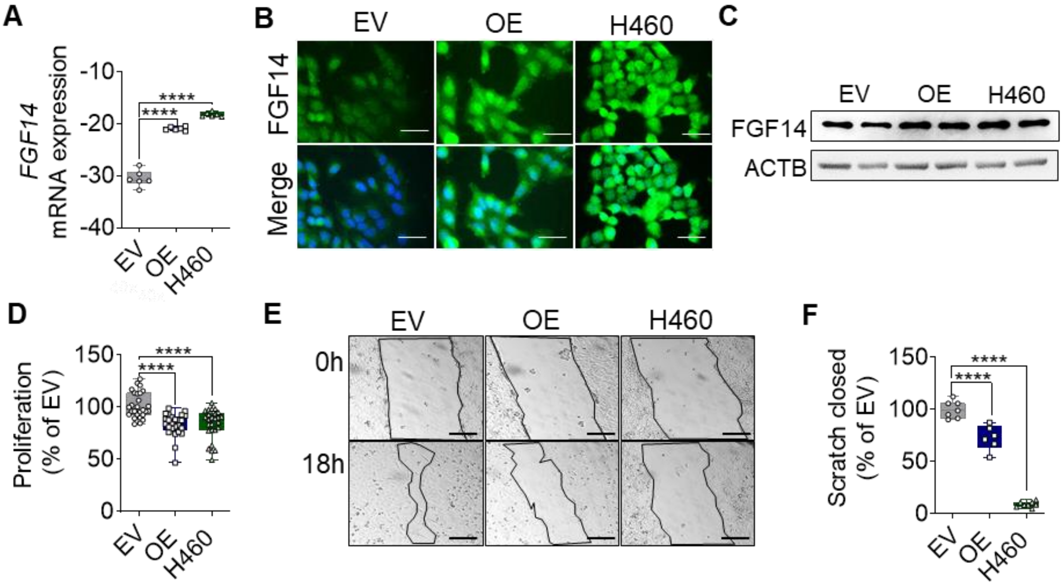

Figure S3. FGF14 expression in H460 cells. Validation of FGF14 expression by H460 parental cells compared with A549-empty vector (EV) and A549-FGF14-expression vector (OE)-transfected cells via (**A**) real-time polymerase chain reaction, (**B**) immunocytochemistry staining, and (**C**) western blot. (**D**) Quantification of protein expression by H460 and H838-FGF14-OE cells compared with A549-EV control cells by relative density normalized to β-actin. (**E**) Cellular proliferation was quantified by bromodeoxyuridine incorporation in H460 and A549-FGF14-OE cells compared with H838-EV control cells. (**F**) The migratory ability of H460 and A549-FGF14-OE cells was evaluated by scratch assay compared to the A549-EV control cells. Representative photographs were taken at 0 and 18 h after scratching (5× magnification). Cells in B were labeled using FGF14 antibody and revealed by an AlexaFlour 488 secondary antibody (green). DNA was stained with 4′,6-diamidino-2-phenylindole (blue), scale bars, 100 μm. Data are shown as mean ± standard error of the mean using a one-way analysis of variance. *P*-values ≤ 0.05 were considered statistically significant for all analyses; ****$p \leq$ 0.0001.

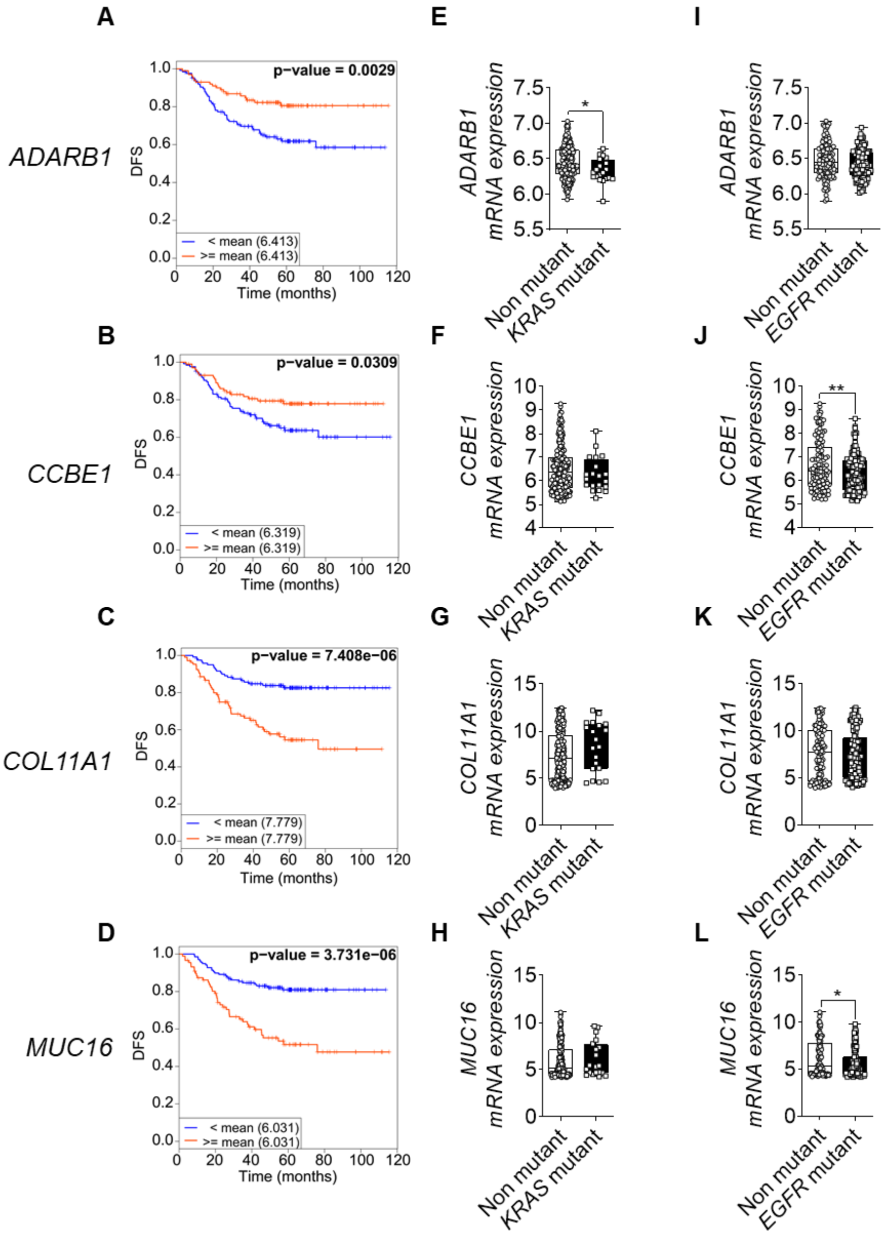

Figure S4. Kaplan–Meier estimate of overall survival in lung adenocarcinoma patients. (**A-D**) Kaplan–Meier estimate of overall survival among Okayama patient dataset with LUAC that are classified according to the m-RNA expression levels of *CCBE1, ADARB1, COL11A1* and *MUC16*: high (above the mean value of mRNA levels) and low (below the mean value of mRNA levels). (**E-H**) Analysis of expression in *KRAS* mutant vs non-mutant patient samples and (**I-L**) *EGFR* mutant vs non-mutant samples. Data obtained from CANCERTOOL. Data are shown as mean ± standard error of the mean using Student's t-test. *P*-values ≤0.05 were considered statistically significant for all analyses. *$p \leq 0.05$ and **$p \leq 0.01$.

Site. Available online: URL (accessed on Day Month Year).

© 2020 by the authors. Submitted for possible open access publication under the terms and conditions of the Creative Commons Attribution (CC BY) license (http://creativecommons.org/licenses/by/4.0/).
